# A Comparison of Impact of Chronic Periodontal Diseases and Nonsurgical Periodontal Therapy on Oral Health-Related Quality of Life

**DOI:** 10.1155/2017/9352562

**Published:** 2017-05-14

**Authors:** Khushboo Goel, Dharnidhar Baral

**Affiliations:** ^1^Department of Periodontology and Oral Implantology, B.P. Koirala Institute of Health Sciences, College of Dental Surgery, Dharan 56700, Nepal; ^2^School of Public Health and Community Medicine, B.P. Koirala Institute of Health Sciences, Dharan 56700, Nepal

## Abstract

**Objectives:**

To evaluate the impact of chronic periodontal diseases (PDs) and compare phases of nonsurgical periodontal therapy (NSPT) on oral health-related quality of life (OHRQoL) in patients attending a tertiary care center of eastern Nepal.

**Materials and Methods:**

Matched for socioeconomic status, participants were recruited in two groups: moderate-to-severe chronic periodontitis (*n* = 24, 43 ± 46 years) and chronic gingivitis (*n* = 25, 30 ± 96 years). The treatment modalities were scaling and root surface debridement (RSD) and supragingival scaling, respectively. The impact of periodontal disease treatment status was assessed by a self-reported questionnaire of Nepali Oral Health Impact Profile (OHIP-14) at baseline and 9–12 weeks after NSPT.

**Results:**

The median (IQR) OHIP-14 total scores for PDs reduced from 7 (3–11) to 3 (1–7.5) after NSPT. Both groups showed a significant improvement on OHRQoL (*p* value < 0.001). The periodontitis group showed an increased median (IQR) reduction of 52% (35.22–86.15) compared with the gingivitis group with 27% (0.00–50.00). The impact on orofacial pain, orofacial appearance, and psychosocial dimensions was observed, which improved after NSPT in both groups.

**Conclusion:**

PDs are directly associated with OHRQoL and treatment of the disease may enhance quality of life from a patient's perspective. Scaling and RSD provided better influence on OHRQoL than supragingival scaling.

## 1. Introduction

There have been dramatic improvements in oral health states in recent decades, but periodontal disease has remained prevalent and with little signs of improvement in the severity of the disease [1]. Periodontal diseases (PDs) cause tooth loss at the end stage of the disease but, ironically at an early stage, no to very few symptoms (swelling, bleeding, and pain) are reported by patients. Therefore, PDs are considered a “silent form of disease” that further impedes tooth preservation [2]. However, recently, their occurrence has been shown to have a substantial sociobehavioral component and, thus, they are considered not only to be a threat to the dentition, but also to affect oral health-related quality of life (OHRQoL) [3]. OHRQoL is defined as a multidimensional build that reflects people's comfort when eating, sleeping, and engaging in social dealings; their self-esteem; and contentment with respect to their oral health [4].

Patient based outcomes are becoming increasingly popular as their opinions differ from true clinical end points of gain in clinical attachment level and decrease in probing pocket depth [5, 6]. Symptoms such as bleeding gums, tooth mobility, drifting teeth, and unaesthetic loss of anterior papilla can be a symbolic oral health-related problem as it can compromise the ability of the periodontal disease person to eat, speak, socialize, and do various daily activities [5]. These patient perspectives can act as significant indicators to be assessed and help identify individual needs to achieve a holistic approach towards oral health care.

The extent of the impacts could be assessed by different OHRQoL measures [7–9]. Oral Health Impact Profile (OHIP-14) is one of the well validated measures of OHQoL [10]. It is considered good at predicting broader psychological well-being and life satisfaction and its sensitivity to change has also been assessed [11]. OHIP-14 can assess the impact of periodontal disease from a patient's perspective and can detect changes in quality of life (QoL) before and after therapy [12]. It is considered to have better internal consistency reliability as it has more items [13] than other measures of OHRQoL [7]. Currently, OHIP-14 has been validated for a wide range of populations in different countries and a translated Nepali version of the short form of OHIP-14 questionnaire was used to measure the QoL in the Nepalese adult population [14]. The impact of periodontitis on OHRQoL measures is well recognized worldwide [6, 12, 15]. It is now, however, important to consider its implementation in clinical practice.

Periodontal treatment may be in the form of nonsurgical or surgical therapy. Change in OHRQoL after surgical therapy shows contradictory reports [16, 17]. Similarly, routine nonsurgical periodontal therapy (NSPT) reports conflicting results of either significant [6, 18] or insignificant improvement [19, 20]. In this part of the world where awareness is limited among people, it is unclear whether periodontal diseases have an influence on their social and psychological well-being. The results are also ambiguous on whether periodontal therapy has a positive effect or whether intervention by a therapist is beneficial for improvement in QoL [20]. Therefore, the aim of this study was to evaluate the impact of NSPT on oral health-related quality of life in periodontal disease patients visiting a tertiary care center in eastern Nepal. Limited data is available over the impact of distinct phases of nonsurgical periodontal treatment [21, 22]; therefore, this study also aims to compare the treatment of nonsurgical periodontal therapy on OHRQoL in gingivitis and periodontitis patients, respectively.

## 2. Materials and Methods

### 2.1. Study Design

This was a comparative cross-sectional study. This research project was approved by the Institutional Ethics Committee of B.P. Koirala Institute of Health Sciences (BPKIHS), Dharan, Nepal (code number IRC/494/015), in accordance with the ethical principles of the Declaration of Helsinki. This is a tertiary health care center with a tertiary dental hospital as well.

### 2.2. Study Setting

This study was carried out at the Department of Periodontology and Oral Implantology, College of Dental Surgery, B.P. Koirala Institute of Health Sciences, Dharan, Nepal.

### 2.3. Criteria for Selection

The Inclusion criteria were as follows: (1) periodontitis group with clinical diagnosis of moderate-to-severe generalized chronic periodontitis, with at least one tooth having pocket depths (PD) ≥ 5–7 mm with ≥3 mm attachment loss in different quadrants (either anterior or posterior), or gingivitis group with clinical diagnosis of generalized chronic gingivitis defined as inflammation of the gingiva with no loss of attachment; (2) presence of at least 16 teeth with exclusion of third molars; (3) no extensive periodontal therapy in the previous 6 months; (4) absence of any known systemic illness; (5) wearing a denture or an orthodontic appliance, having caries or other oral or systemic diseases, taking multiple medications, or any adverse habits.

Exclusion criteria were as follows: (1) diagnosed case of aggressive periodontitis, (2) pregnant and lactating females, (3) smoking, and (4) refusal to provide informed consent.

### 2.4. Sample Size and Sampling Method

The ideal sample size to ensure adequate power for this study was calculated considering the mean for group before and after treatment as 41.08 and 27.68 and standard deviation as 6.80 and 6.93, respectively, with true difference of 1. Based on the above values and using a formula to estimate the sample size of two means, this study considered a total of fifty participants necessary to provide 80% power at 95% confidence interval (*α* = 0.05).

### 2.5. Methods of Data Collection

**Figure 1:**
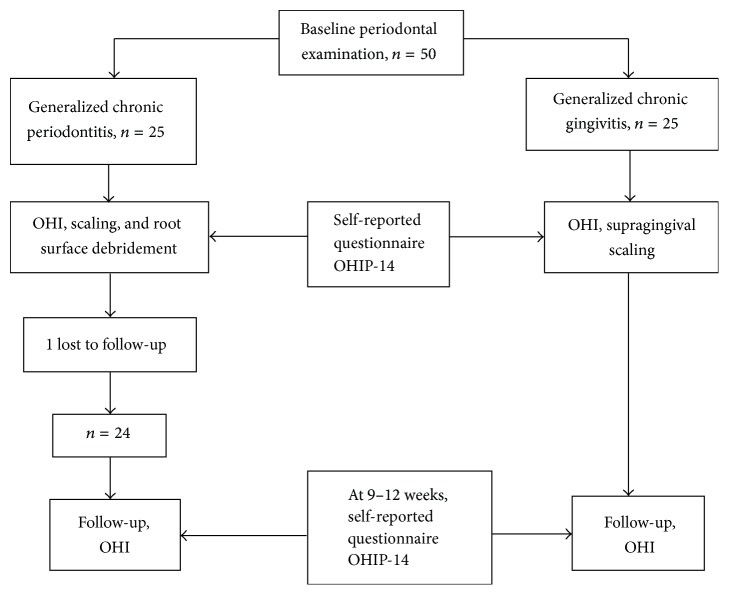
Flow chart of study. OHI: oral hygiene instructions; OHIP-14: Oral Health Impact Profile-14.

One periodontist performed the comprehensive periodontal examination under artificial light with the help of a mouth mirror and periodontal probe (University of North Carolina-15, Hu-Friedy Instruments, Chicago, IL, USA). After baseline examination and necessary tooth extractions, a total of 50 participants were enrolled in this study for both groups. Written consent was obtained from all the participants.  Figure 1 displays the study design and the flow of subjects (Figure 1).

Data was collected using an ordered questionnaire which contained information about sociodemographic characteristics. This included age, sex, frequency of brushing, and previous dental visits. Socioeconomic status included information about education, occupation, and family income per month (in Rs) which is a modification of Kuppuswamy's Socioeconomic Status Scale in context to Nepal [23]. There are different approaches to measure OHRQoL; the most popular one is multiple item questionnaires. To measure the impact of periodontal diseases on the quality of life, a psychosocial instrument questionnaire of short form of Nepali version of Oral Health Impact Profile-14 (OHIP-14) item score was used. For usage in Nepalese populations, the OHIP-14 was translated from the original English version and validated in Nepali. It consisted of a set of 14-item scores with high reliability of Cronbach's alpha value of >0.83 and validity obtained by doing factorial analysis of the scale. This short form of the original 49-item score [10] provides slightly less information but involves less administration and was found to be as reliable as the original questionnaire [14]. The original publication of the OHIP also categorized items into seven domains and named them as Functional Limitations, Physical Pain, Psychological Discomfort, Physical Disability, Psychological Disability, Social Disability, and Handicap and the 14 questions were two from each of the seven domains [10, 24]. These domains were conceptually based on the model of oral health and expert opinion rather than on statistical procedures such as the exploratory factor analysis (EFA) [25]; therefore, John et al. in 2014 evidenced a more differentiated four-dimensional structure of OHIP, namely, oral function, orofacial pain, orofacial appearance, and psychosocial impact, which is similar across cultures and populations [26]. However, one of the important factors in deciding which of the systems should be used is its validation for the population where the study is to be conducted. Therefore, the Nepali Oral Health Impact Profile (OHIP-14) was used in a questionnaire format and these questions were discussed based on the four-dimensional structure of OHIP. Questions were answered on a Likert scale from 0 to 4, with 0 = never, 1 = hardly ever, 2 = occasionally, 3 = fairly often, and 4 = very often. The summary score will range from “0” to a maximum of “56.” “0” represents no problems while higher scores represent poor or impaired oral health-related quality of life.

OHIP-14 was self-completed by all participants of both groups at baseline. Both groups received oral hygiene instructions (OHI) at baseline. The periodontitis group received full mouth scaling and root surface debridement (RSD). Supragingival calculus was defined as calcified deposits that are located on the exposed crown and root surfaces and that extend up to 1 mm below the free gingival margin. An ultrasonic device (Cavitron, Dentsply, York, PA) was utilized to remove supragingival calculus in the first session, and instructions were provided on correct tooth brushing and the use of interproximal brushes. After two weeks, subgingival scaling and RSD were performed in two appointments with the help of hand curettes (Gracey Curettes, Hu-Friedy Instruments, Chicago, IL) and ultrasonic instruments to obtain smooth root surfaces. The gingivitis group underwent full mouth supragingival scaling up to 1 mm beyond the gingival margin with the help of ultrasonic instruments and corrections of plaque retentive margins. Participants were reinforced with OHI appropriate to their needs at recall visits. Approximately 9–12 weeks after the last periodontal treatment, both groups were reviewed and the questionnaire of OHIP-14 items was provided to all, which was self-completed. Appropriate periodontal treatment, including periodontal surgery, was prescribed for those individuals who still had sites with residual PD ≥ 6 mm with bleeding on probing.

### 2.6. Statistical Analysis and Software Used

Only participants completing the questionnaires both at baseline and at the 9–12-week follow-up were included for statistical analysis. Collected data were entered into MS Excel 2007 and converted into the statistical software package SPSS 11.5 (SPSS, Chicago, IL, USA) for statistical analysis. Descriptive statistics like percentage, mean, SD, median, and IQR were calculated along with graphical and tabular presentation made. For inferential statistics, chi-squared test, Mann–Whitney *U* test, and Wilcoxson's signed-rank test were applied to find out the significant difference between groups and within the group. The level of significance was considered ^*∗*^*p* < 0.05 at 95% CI.

## 3. Results

This study comprised 49 adults with a mean age of 37 ± 9.03 (17–60 years) years. Their sociodemographic characteristics (SDC) are shown in Table 1.

The results concerning the impact of periodontal disease on OHRQoL are presented in Table 2. “Never” was reported in 10 out of 14 items of OHIP-14 by more than half of the patients before periodontal therapy. 13 out of 14 items of OHIP-14 reported “never” after NSPT. There were less reported answers “very often” from both groups. This indicated an impact of periodontal diseases on certain items of OHIP questionnaire and an improvement seen after the periodontal therapy in respective items and simultaneously in their QoL. The median (IQR) OHIP-14 total score at baseline for both groups was 7 (max. = 22, min. = 0, IQR = 3–11). This decreased slowly following the completion of nonsurgical periodontal treatment, scoring a median of 3 (maximum = 16, minimum = 0, IQR = 1–7.5). This indicated poor QoL of periodontal disease patients at baseline that improved significantly after periodontal treatment at 9–12 weeks (*p* value < 0.001) (Table 3). The overall median (IQR) reduction in percentage was found to be 46.6 (20.0–73.21) in periodontal disease patients. There was a significant difference between the groups at baseline (*p* value = 0.015) but the difference was reduced after periodontal therapy (*p* value = 0.747) (Table 4). Both groups showed a statistically significant improvement in the score of OHIP-14 before and after treatment (*p* value = 0.001) (Table 5). The periodontitis group after scaling and RSD showed increased median (IQR) reduction in percentage, 51.9 (35.22–86.15), compared to median (IQR) percentage reduction in the gingivitis group after supragingival scaling, 27.2 (0.00–50.00). The reduction in percentage was significant between the groups.

## 4. Discussion

This study comprised 49 adults with a mean age of 37.0 ± 9.03 years. This study showed a significant impact of periodontal diseases on OHRQoL. The result of this study is in agreement with findings of most of the other studies [2, 6, 27–30] showing poorer QoL in periodontal disease individuals. This study compared the effect on change in OHRQoL using OHIP-14 score before and after NSPT. The results were statistically significant to show enhanced improvement after receiving the therapy in periodontal disease individuals. The findings were similar to other studies showing notable improvement [6, 16, 22, 27, 31]. Median (IQR) OHIP-14 scores in this study reduced from 7 (3–11) at baseline to 3 (1–7.5) 9–12 weeks after treatment (*p* value < 0.001). These scores of median reduction are comparable with the findings reported in different populations of periodontal disease patients worldwide [6, 18, 31].

The assessment of NSPT is generally made in no less than 1 to 3 months; therefore, in this study, OHRQoL was analyzed after 9–12 weeks [32]. No adjunctive therapies to scaling and RSD were compared in this study. A recent meta-analysis has shown that scaling and root planning result in 0.5 mm improvement in clinical attachment level (CAL) at a moderate level of certainty against 0.2 to 0.6 mm improvement in CAL in combination with adjuncts [33]. A Nepali version of OHIP-14 item score was used. The OHIP was designed to provide a comprehensive measure of oral functional limitations, oral pain, and discomfort and the psychological and behavioral impacts of oral conditions [24], and currently with the exploratory factor analysis of Oral Health Impact Profile there is evidence for a more differentiated four-dimensional structure of OHIP items. It is one of the most widely used instruments and has been used to assess the impact of periodontal disease as well [5, 6, 18]. The OHIP-14 item scores were significantly associated with periodontal symptoms which include swollen, sore, or receding gums, toothache, loose teeth, and bad breath [5].

In this study, the impact of chronic periodontal diseases and their particular treatment were compared before and after NSPT. The periodontitis group had higher mean age of 43.46 ± 7.6 years compared to the gingivitis group. All other sociodemographic data were similar with participants belonging to the upper middle socioeconomic class. Healthy controls were not included in this study as it was expected that they would have better OHRQoL than diseased individuals. A study done by Jowett et al. [6] in 2009 compared between two cohorts with periodontal disease but it was done only for 7 days and nonsurgical phases of treatment were not compared. In this study, there was a significant difference between the groups at baseline (*p* = 0.015). This can be explained by the fact that, in the periodontitis group, the disease symptoms were more severe regarding pain, mobility, unesthetic loss of papilla, greater probing depths, and bleeding gums as compared to the gingivitis group that reported very few of these impacts. Both groups showed improvement after periodontal therapy on OHRQoL; however, the treatment done by scaling and RSD showed increased median (IQR) reduction in percentage, 51.92 (35.22–86.15), compared with the gingivitis group after supragingival scaling, 27.27 (0.00–50.00). Studies that compare both of these treatments separately are scarce, but recently a study done by Mendez et al. in 2016 [22] showed improvement after supragingival treatment, but they did not describe RSD as a separate phase of treatment. The periodontitis group had higher median (IQR) OHIP-14 score signifying poor QoL. The findings of this study are comparable to studies showing that extent/severity affects QoL [5, 12, 34] and greater improvement is seen in patients with greater severity [2, 6, 18, 35].

Researchers consider the change in oral health to be assessed by one score as meaningful, but periodontal diseases are complex diseases and there is a danger in the interpretation of the results as one because one aspect may have improved and another might have deteriorated [36]. Pain in the mouth was not reported “very often” by both groups, but 40.8% of the patients reported “occasional pain” initially; after treatment, only 10.2% of the patients reported pain. This signified an improvement perceived by patients and is in agreement with most of the studies showing improvement after periodontal therapy [18, 31]. Significant improvement in pain was also observed in OHRQoL measures other than OHIP-14 [37–39]. Although a reduction in the frequency of problems of orofacial pain and improvement in clinical signs of periodontal pathology can be expected after NSPT, there are other inevitable outcomes of periodontal therapy such as cervical sensitivity, gingival recession, pathologically migrated teeth, and loss of papillae which may affect a patient's appearance as well as having an impact psychosocially. In the periodontitis group, 37.4% of the patients were irritable with others that ranged from a response of “hardly ever to very often” that reduced to 24.9% after treatment and 58.3% of patients were embarrassed in front of other people that improved to 41.7% after treatment. Marked differences were not observed in these aspects in periodontitis patients as sometimes NSPT might not be enough for treatment but a tendency to improvement was seen. This signified that chronic periodontitis may interfere with psychosocial aspects of periodontal disease patients and therefore we may challenge the perception of chronic periodontitis as a silent disease [40]. However, in the gingivitis group, patients had less severe symptoms and complained mostly of enlarged gums, malodor, and bleeding. These symptoms were reduced after treatment and improvements were appreciable by the patients. Also, in the periodontitis group, 79.2% of the patients were conscious of their appearance, with 75.1% feeling tensed, which improved to 45.8% and 58.4% after treatment, respectively. In the gingivitis group, 52.0% of the patients were conscious, with 68.0% feeling tensed, which improved to 36.0% and 44.0%, respectively. It may be therefore predicted that orofacial appearance has a definite impact on quality of life of periodontal disease patients and frequent visits and/or intervention by the therapist might have provided them with some form of gain in confidence and positive feeling. The results are comparable to other studies showing similar results [18, 31].

The oral function was not affected to a great extent as most of the patients reported “never” for difficulty in speech (75.5%) and altered taste (85.7%) in both groups. Findings are comparable to studies demonstrating the least improvement in this dimension [18, 31]. The reason might be that patients were dentulous and only a few patients reported discomfort with mobility. Aggressive periodontitis patients that can be expected to have poorer QoL and affected functionality were also not included [5, 41]. Changes in different dimensions may be affected by the signs and symptoms reported but clearly both treatment protocols provided improved periodontal health and helped to bring about a change in an individual's QoL. The participants in this study were of a younger age group. This may prove that even younger individual's QoL is affected, which might improve after therapy.

One of the major limitations of this study is the high prevalence of subjects with zero scores which may compromise the ability of OHIP-14 to detect within-subject changes. Nevertheless, Nepalese language validation of other measures such as condition specific OHQoL tool, GOHAI (General Oral Health Assessment Index), and OHQoL-UK (Oral Health Quality of Life-UK) which reports positive effect of oral conditions and not just frequency is highly desirable. Further long-term studies with larger populations are required to address the above limitations.

In conclusion, this study clearly indicated that periodontal diseases negatively impact an individual's quality of life in many aspects, especially of those patients suffering from chronic generalized periodontitis compared to patients with gingivitis. Nonsurgical periodontal therapy has a constructive role to play to ameliorate the impact, with significant changes observed for full mouth scaling and root surface debridement procedure.

## Figures and Tables

**Table 1:** Association between groups and related SDC.

Characteristics	Category	Total number (%)	Number (%)		*p* value
			Periodontitis	Gingivitis	
Age group in years	>29	9 (100.0)	0 (0.0)	9 (100.0)	—
	30–39	18 (100.0)	5 (27.8)	13 (72.2)	
	40–49	17 (100.0)	14 (82.4)	3 (17.6)	
	>50	5 (100.0)	5 (100.0)	0 (0.0)	

Mean age in years ± SD		49 (100.0)	43.46 ± 7.6	30.96 ± 6.4	0.001^*∗*^

Gender	Female	23 (46.9)	12 (52.2)	11 (47.8)	0.674
	Male	26 (53.1)	12 (46.2)	14 (53.8)	

Brushing frequency	1	33 (67.3)	18 (54.5)	15 (45.5)	0.263
	≥2	16 (32.6)	6 (37.5)	10 (62.5)	

Socioeconomic status	Upper middle	24 (49.0)	11 (45.8%)	13 (54.2%)	0. 736
	Lower middle	15 (30.6)	7 (46.7%)	8 (53.3%)	
	Upper lower	10 (20.4)	6 (60.0%)	4 (40.0%)	

^*∗*^Statistically significant *p* value (^*∗*^*p* < 0.05).

**Table 2:** Distribution of responses to OHIP-14 item scores for all participants.

OHIP-14 item questions	Before, *n* (%)					After, *n* (%)				
	0	1	2	3	4	0	1	2	3	4
Trouble pronouncing words	37 (75.5)	5 (10.2)	5 (10.2)	2 (4.1)	0.0	39 (79.6)	6 (12.2)	4 (8.2)	0.0	0.0
Taste has worsened	42 (85.7)	5 (10.2)	2 (4.1)	0.0	0.0	46 (93.9)	2 (4.1)	1 (2.0)	0.0	0.0
Pain in mouth	18 (36.7)	7 (14.3)	20 (40.8)	4 (8.2)	0.0	32 (65.3)	12 (24.5)	5 (10.2)	0.0	0.0
Uncomfortable eating food	39 (79.6)	3 (6.1)	5 (10.2)	2 (4.1)	0.0	42 (85.7)	4 (8.2)	3 (6.1)	0.0	0.0
Self-consciousness	17 (34.7)	11 (22.4)	11 (22.4)	9 (18.4)	1 (2.0)	28 (57.1)	12 (24.5)	5 (10.2)	3 (6.1)	1 (2.0)
Tense feeling	14 (28.6)	10 (20.4)	14 (28.6)	7 (14.3)	4 (8.2)	20 (40.8)	14 (28.6)	10 (20.4)	4 (8.2)	1 (2.0)
Unsatisfactory diet	47 (95.9)	1 (2.0)	0.0	1 (2.0)	0.0	48 (98.0)	0.0	1 (2.0)	0.0	0.0
Interruption of meals	46 (93.9)	2 (4.1)	0.0	1 (2.0)	0.0	46 (93.9)	1 (2.0)	2 (4.1)	0.0	0.0
Difficulty relaxing	32 (65.3)	9 (18.4)	4 (8.2)	3 (6.1)	1 (2.0)	39 (79.6)	7 (14.3)	2 (4.1)	1 (2.0)	0.0
Feeling embarrassed	22 (44.9)	11 (22.4)	9 (18.4)	5 (10.2)	2 (4.1)	27 (55.1)	12 (24.5)	6 (12.2)	4 (8.2)	0.0
Irritable with others	33 (67.3)	6 (12.2)	3 (6.1)	4 (8.2)	3 (6.1)	38 (77.6)	2 (4.1)	5 (10.2)	4 (8.2)	0.0
Difficulty doing usual jobs	47 (95.9)	2 (4.1)	0.0	0.0	0.0	49 (100.0)	0.0	0.0	0.0	0.0
Less satisfaction	47 (95.9)	1 (2.0)	1 (2.0)	0.0	0.0	47 (95.9)	1 (2.0)	1 (2.0)	0.0	0.0
Totally unable to function	49 (100.0)	0.0	0.0	0.0	0.0	49 (100.0)	0.0	0.0	0.0	0.0

0: never; 1: hardly ever; 2: occasionally; 3: fairly often; 4: very often.

**Table 3:** Association of periodontal diseases OHIP-14 item score before and after NSPT^#^.

Test	Median score (IQR)
Before	7 (3–11) (0–22)
After	3 (1–7.5) (0–16)

^#^Wilcoxon's signed-rank test.

**Table 4:** Association between the groups of OHIP-14 item score before and after NSPT^†^.

Test	Median (IQR)		*p* value
	Periodontitis (scaling and RSD)	Gingivitis (supragingival scaling)	
Before	8.50 (4.25–15.00)	4.00 (2.50–10.00)	0.015^*∗*^
After	3.00 (1.00–7.50)	2.00 (1.00–7.50)	0.747

^†^Mann–Whitney *U* test. ^*∗*^Statistically significant *p* value (^*∗*^*p* < 0.05).

**Table 5:** Association between intragroups of OHIP-14 item score before and after NSPT^‡^.

Group	Median (IQR)		*p* value
	Before	After	
Periodontitis	8.50 (4.25–15.00)	3.00 (1.00–7.50)	<0.001^*∗*^
Gingivitis	4.00 (2.5–10.00)	2.00 (1.00–7.50)	0.001^*∗*^

^‡^Wilcoxson's signed-rank test. ^*∗*^Statistically significant *p* value (^*∗*^*p* < 0.05).

## Additional Points

The clinical efficacy after periodontal treatment is irrefutable, but along with the treatment provided, if the clinicians can help understand the patient's perspective in a better way, it can definitely provide a holistic approach towards patient's oral care.
